# The Burden of Routine in Mental Hospitals

**Published:** 1913-11-29

**Authors:** 


### THE BURDEN OF ROUTINE IN MENTAL HOSPITALS.

### Its Limiting Effect on Research.

There is rather a marked propensity among alienists to overlook preventive measures whilst attempting to deal with the products which the adoption of those measures might have obviated. It is very well to manufacture tuberculin in large quantities; it were better to obviate by right methods of hygiene the necessity for the use of that tuberculin which the most ardent advocates of its employment would hardly designate as a tonic! And so with insanity. Would it not be well to make more adequate provision for research into the causation of the morbid brain states which underlie the conditions designated—with rather a mixture of metaphysical and material nomenclature—mental diseases? Upon what, for instance, does the condition designated as paranoia, or systematised persecutionary insanity, depend—in what convolutions is the morbid process? How does premature dementia arise? What are the causes of melancholia or of mania? And so on in many other cases. Is the disorder primarily in the nerve-cells or is it secondary to some bodily condition or some toxæmia? There is no lack of theories in regard to these matters, but what is required is that, if possible, these theories shall be verified by researches into the pathology of cerebral conditions.

It will be readily admitted that there is no lack of clinical material; the figures in the annual reports of the Commissioners in Lunacy make that only too clear. The difficulty lies in the fact that the labourers in the field of cerebral pathology are too few; and the question is rather as to how this dearth of workers is to be overcome. The scarcity of workers is, however, probably dependent upon economic considerations. There has been a decided tendency of late for young practitioners to fight shy of appointments in mental hospitals, and the reason for this is not far to seek. It has been realised that the salaries are inadequate; or that, if not inadequate at the commencement of the work, the prospects for the future are uninviting. The authorities are realising this, with the result that steadily there is an advance in the amount of the salaries offered.

So far so good; but assistant medical officers in asylums are so greatly taken up with routine work in the institution that they are likely to have little opportunity of doing research work, and even when opportunity arises their energies, absorbed chiefly by these routine duties, will probably prove insufficient for prolonged investigations. There are exceptions to this, and honourable ones, but they are too few to disprove the rule.

It has been said that the question is largely, if not entirely, an economic one. Provided that the means are found to pay reasonable salaries it is practically certain that the men will soon be found to work efficiently as specially appointed pathologists in the large mental hospitals in conjunction with the other members of the medical staff. The financial inducements must, however, be adequate, otherwise the best men will not turn towards this gravely important region of pathological research. There has been, however, for so many years an eleemosynary tendency on the part of the public towards the medical profession that such a suggestion as that the labourer, if he is a medical man, is worthy of his hire is still frequently received with disapprobation. As it is becoming increasingly difficult for the medical man to live by praise alone, as Huxley expressed it, the necessity of adequate remuneration even for scientific work grows more and more apparent. Certainly the difficulties in the way of such appointments are not insuperable; and the results of the investigations of those appointed might soon justify the additional expense by making the treatment of insanity less empirical and more rational, as the suggested causation of the morbid process is steadily removed from the region of specious theory into that of acknowledged fact.

In the study of the integration of the nervous system much fine and useful work has been done, and many valuable therapeutic results have accrued. The process of disintegration remains largely to be investigated, and there is little doubt but that equally beneficial consequences may issue from research in that direction.

